# British Heart Foundation Data Science Centre 2.0: faster, fairer cardiovascular data-driven discovery

**DOI:** 10.1093/eurheartj/ehag416

**Published:** 2026-06-05

**Authors:** Steffen E Petersen, Rhoswyn A Walker, James Leiper

**Affiliations:** William Harvey Research Institute, NIHR Barts Biomedical Research Centre, Queen Mary University London, Charterhouse Square, London, EC1M 6BQ, UK; Barts Heart Centre, St Bartholomew's Hospital, Barts Health NHS Trust, West Smithfield, EC1A 7BE, London, UK; Health Data Research UK, Gibbs Building, 215 Euston Road, NW1 2BE, London, UK; Health Data Research UK, Gibbs Building, 215 Euston Road, NW1 2BE, London, UK; British Heart Foundation, The Cube, 43a Leith Street, Edinburgh EH1 3AT, UK

**Data now sit at the centre of cardiovascular discovery and care. Building on 5 years of delivery, the British Heart Foundation (BHF) Data Science Centre (DSC) is evolving into an open, comprehensive, service-oriented model to modernize the UK’s cardiovascular data ecosystem. ‘BHF DSC 2.0’ will provide user-friendly discovery and access tools, reusable research-ready data products, expert linkage and curation, and proportionate information-governance advice, acting as a national core facility aligned with the future ambitions of the Health Data Research Service. Services will scale across four priority domains: national datasets; consented cohorts and trials; high-resolution regional data (including electrocardiograms); and cardiovascular imaging. The program will accelerate research impact—from AI-enabled diagnostics to pragmatic trials—while strengthening transparency, public involvement, and UK–European collaboration, including alignment with the European Society of Cardiology’s Data Science Committee and opportunities under the European Cardiovascular Health Plan. The destination is clear: dependable expert data services that turn ideas into insights, and insights into patient benefit.**

## Why data, and why now?

Cardiovascular research is at a tipping point: advances in storage, linkage, and analytics mean we can answer clinical questions at a population scale and with new precision—if data are findable, accessible, interoperable, and reusable. BHF’s early investment created the Data Science Centre (DSC, https://bhfdatasciencecentre.org) to unlock the UK’s health data potential for cardiovascular science; this foundation now supports a step-change in how the community discovers, accesses, and uses data responsibly.

## What changes in BHF DSC 2.0?

The centre is moving to a service-oriented model designed with and for users. Its services will be open to all and designed with and for the cardiovascular research community. Three promises underpin the offer: Tools and interfaces that make discovery and safe access simpler; reusable research-ready data products built with high-quality, reproducible engineering; and expert advice on access, linkage, curation, and information governance.

This is not a new data repository, but a UK-wide service that will work in close partnership with a range of data custodians to facilitate, curate, and engineer data where they already sit—streamlining routes through existing trusted environments and reducing duplication of effort. The goal is faster, fairer, and more impactful research, supported by transparent processes and sustained public involvement.

## A clear 4 × 4 service architecture to deliver ambitions

**Figure 1:**
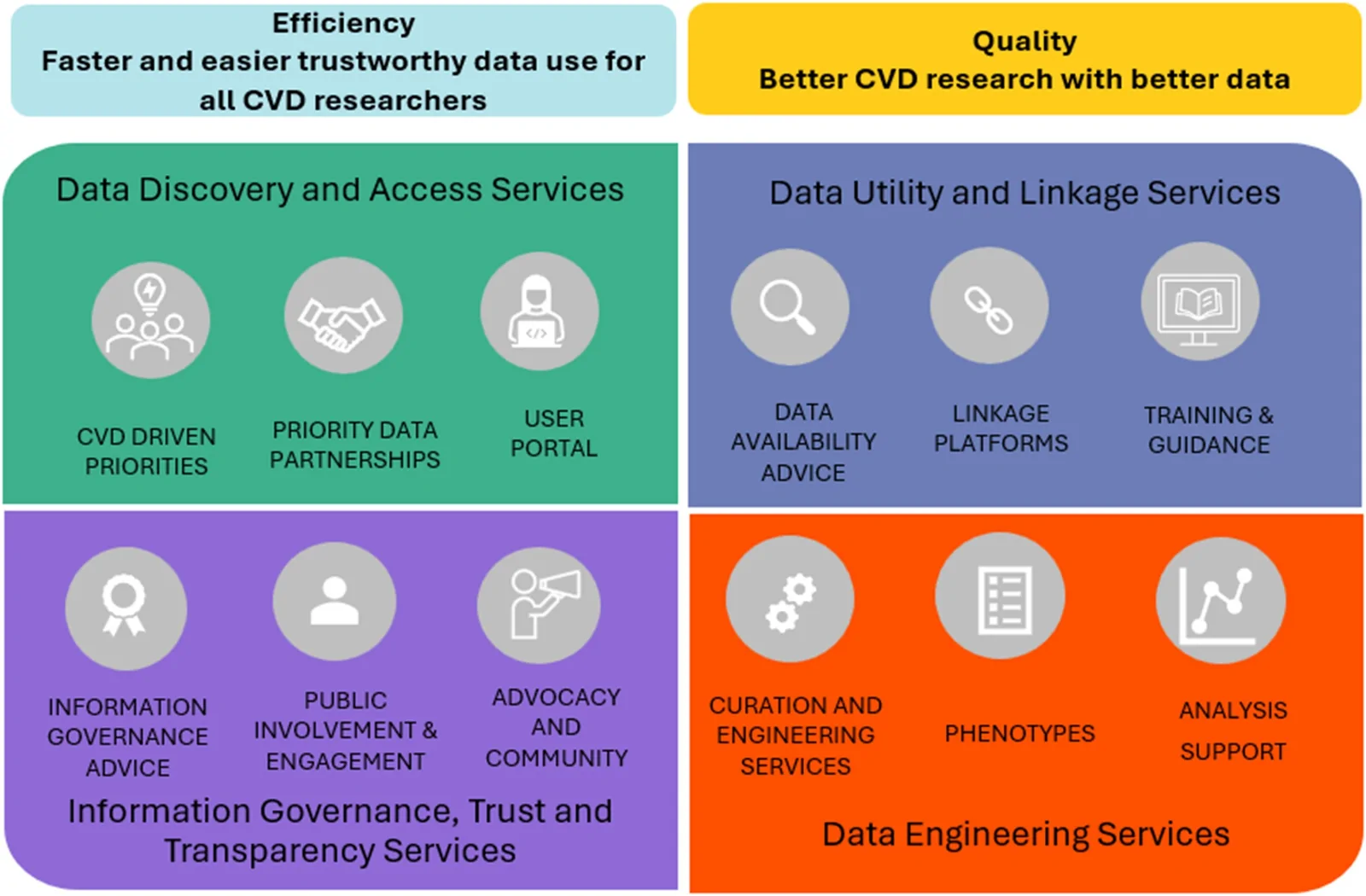
BHF DSC 2.0 objectives and service architecture

*Figure 1* shows how two overarching goals—efficiency (faster, easier, and trustworthy data use) and quality (better cardiovascular research with better data)—are delivered through four service areas: (i) data discovery and access, (ii) information governance, trust, and transparency, (iii) data utility and linkage, and (iv) data engineering. These services support four priority domains: national datasets, consented cohorts and trials, high-resolution regional data (including electrocardiograms), and cardiovascular imaging. The model underpins user-centred tools, research-ready data products, and expert advice to modernize the UK cardiovascular data ecosystem.

## From siloed assets to research-ready resources

The centre will focus on unlocking high-value, underused datasets—imaging, electrocardiograms, and multimodal clinical data—while continuing to add value to national assets across all four nations in the United Kingdom. The data strategy follows clear principles: make the simple easy, make the difficult possible, get ready for the future, and leverage partnerships—consistently applying FAIR (Findable, Accessible, Interoperable, Reusable) standards. BHF DSC 2.0 will act as a national core facility for cardiovascular researchers, aligned with the future ambitions of the £600 million Health Data Research Service, ensuring consistent, navigable access and curation, specifically tailored for the whole cardiovascular research community.

## Practical help that shortens the time to insight

Researchers will see tangible support: data-availability dashboards and feasibility checks, reusable curation code and phenotypes, and hands-on help us to navigate existing data and linkage platforms (e.g. NHS England’s Secure Data Environment network, SAIL in Wales, Scotland’s Safe Haven, and Northern Ireland’s Honest Broker). A flagship example is UK Clinical Cohorts (UK CliC)—a secure platform that links disease-specific cohort data to UK-wide electronic health records, with streamlined governance, curated data, and pathways for future multimodal inputs (imaging, omics, and wearables) and clinical trials. Early adoption spans dozens of cohorts across many institutions, with secondary-use routes built in to maximize scientific return.

## What will this enable?

The service model is designed to power: data-driven prevention, using artificial intelligence (AI) for early detection and risk stratification; scalable, research-ready assets that speed up trials and real-world studies; AI-catalysed innovation across diagnostics, therapeutics, and digital tools; consistent data standards that raise quality and interoperability; and global partnership, aligning UK assets with European and international initiatives.

## Alignment with the ESC and the European Cardiovascular Health Plan

BHF DSC 2.0’s priorities align with the European Society of Cardiology (ESC) Data Science Committee’s ambitions. The Centre’s service stack—particularly research-ready products, linkage platforms, and governance support—directly serves these ambitions. There is a strong pipeline for European collaboration. Through HDR UK networks, the centre will champion sharing methods, metadata, and phenotypes to enable cross-national cardiovascular research. These links map neatly to opportunities within the European Cardiovascular Health Plan to scale AI-enabled diagnostics, embed pragmatic trials in routine care, and integrate imaging, biosignals, and molecular data for precision prevention.

## Designed around users and trust

Services are co-created with BHF leadership, an expert Advisory Group and data partners, and will be iteratively expanded as capabilities mature. National datasets and consented clinical data are ready early; higher-resolution regional data and imaging will be phased in as pipelines, standards, and validation are completed. Public involvement and clear routes for feedback remain embedded to protect social licence and improve equity of access.

## The bottom line for clinicians and researchers

For clinicians, BHF DSC 2.0 means faster answers to service-relevant questions, from risk prediction to outcomes and quality improvement. For researchers, it means one front door to dependable curation, linkage, and analysis support—reducing time from idea to insight and increasing reproducibility. In short: a national, user-centred service that modernizes UK cardiovascular data and connects it purposefully to European efforts for the benefit of people living with cardiovascular disease.

